# A new wing skeleton of the Jehol tapejarid *Sinopterus* and its implications for ontogeny and paleoecology of the Tapejaridae

**DOI:** 10.1038/s41598-022-14111-2

**Published:** 2022-06-17

**Authors:** Chang-Fu Zhou, Dongxiang Yu, Ziheng Zhu, Brian Andres

**Affiliations:** 1 College of Earth Science and Engineering, Shandong University of Science and Technology, Qingdao, Shandong Province China; 2 Department of Health, University College Birmingham, Birmingham, UK

**Keywords:** Palaeontology, Palaeontology

## Abstract

The tapejarid pterosaurs flourished in the Jehol Biota with an abundance of immature individuals and a rarity of individuals at skeletal maturity. Most of these individuals plot well on an ontogenetic series based on the proportions of limb elements, but this has lacked histological evidence until now. Here, a new wing skeleton of *Sinopterus* was thin-sectioned to provide the first histological data about the ontogeny of the Jehol tapejarids. Histologically, the new specimen is an immature individual at a late juvenile stage prior to sexual maturity. It is grouped with medium-sized and medium-crested individuals, which are distinct from the small-sized and crestless individuals as well as the rare large-sized and large-crested individuals at skeletal maturity, supporting the presence of the premaxillary crest as an ontogenetic feature in the Jehol tapejarids. Furthermore, this histology indicates that the largest skeletally immature individuals might have reached the sexual maturity. Enigmatically, there is a size gap between sexual and skeletal maturity, which is at about 79% of the large size, implying a ontogenetic strategy comparable with *Pteranodon* and possibly with the Brazilian tapejarid *Caiuajara*. This size gap is consistent with lack of the larger sexually mature individuals in the Jehol Biota, which is hypothesized to be a migratory habitat for the Jehol tapejarids.

## Introduction

The Tapejaridae are a notable azhdarchoid clade of pterosaurs, characterized by a prominent premaxillary crest and huge nasoantorbital fenestra^1^. This group is widely distributed in the Early Cretaceous, being found in Africa, Asia, Europe, and South America^2–5^. They flourished in the Jehol Biota with a preponderance of immature individuals^1,6,7^. However, these immature fossils were likely subject to taxonomic misinterpretations, resulting in nine nominal species^1,7–14^. The putative diagnostic features (e.g., cranial crest and limb proportions) of these species are possibly the result of individual variation, ontogeny, or sexual dimorphism as evident in *Caiuajara*^15^ and other pterosaurs (e.g., *Anhanguera*, *Hamipterus*, *Pteranodon*, *Pterodactylus,* and *Tupuxuara*)^16–20^. Here, we provide the first study of the histology of the Jehol tapejarids, shedding a new light onto the ontogeny and paleoecology of this group.

The ontogenetic histology of several pterosaurs has been documented (e.g. *Pterodaustro*^21,22^, *Rhamphorhynchus*^23^). They have a fast growth rate at an early ontogenetic stage that is characteristic by the highly vascularized fibro-lamellar bone. Lasting for two to three years, the growth rate then becomes slower with parallel-fibred to laminar bone deposited (e.g., fully-developed endosteal lamina), which is widely accepted as the onset of the sexual maturity^21,22,24,25^. Here, we describe a new wing skeleton of the Jehol tapejarid *Sinopterus* from the Jiufotang Formation of Sihedang Township, Lingyuan City, western Liaoning Province. It is medium-sized, well grouped with the crested individuals, and separated from the small and crestless individuals as well as the rare skeletally mature adults. It is possible that this is a late juvenile ontogenetic stage prior to sexual maturity, characteristic by highly vascularity, a single line of arrested growth (LAG), secondary osteons, and locally developed endosteal laminae. Based on this histology, we discuss the ontogeny, paleoecology, and migratory habit of the tapejarids.

## Results

### Material and geological setting

**Figure 1:**
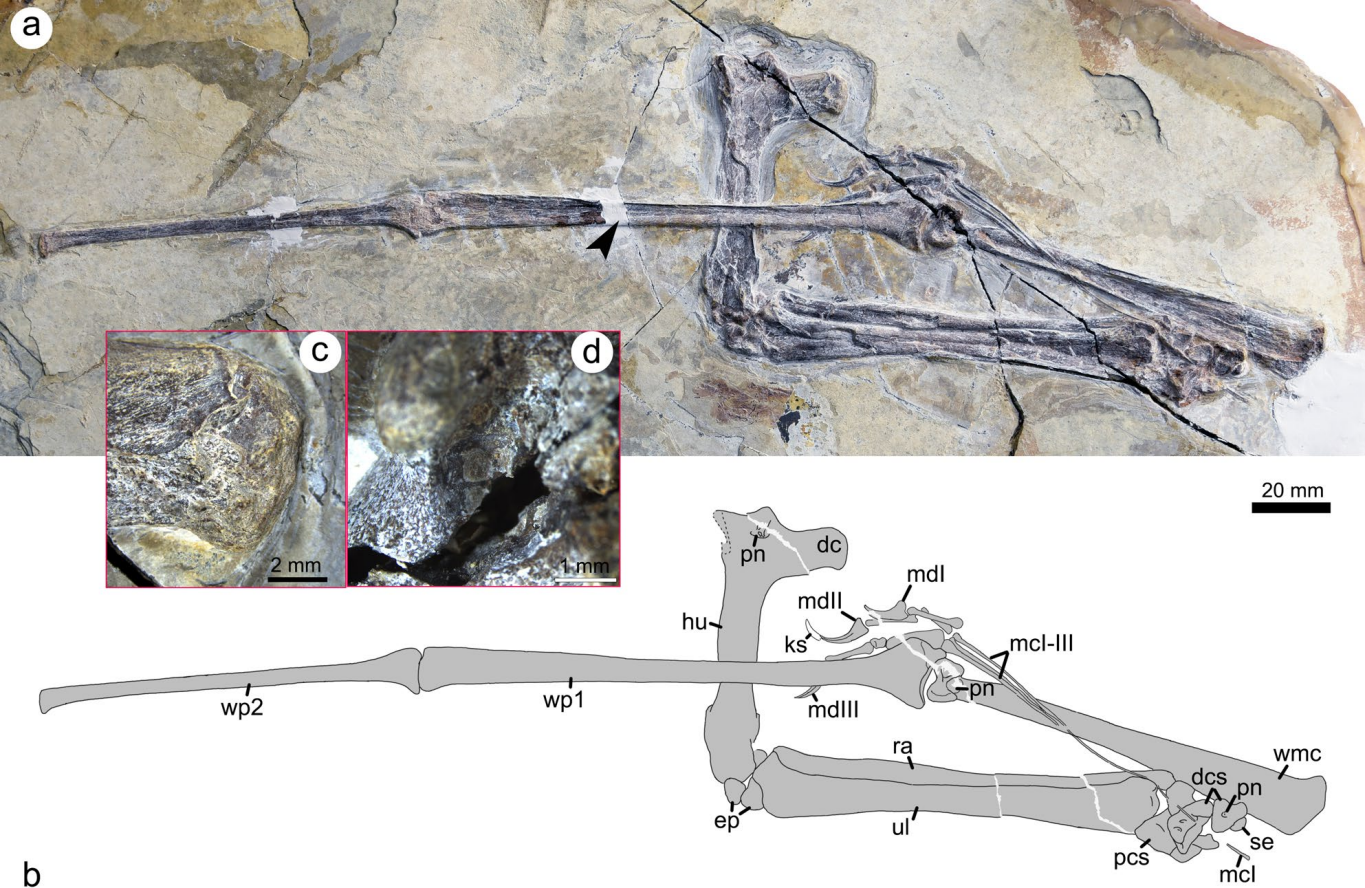
The Jehol tapejarid *Sinopterus* (SDUST-V1014). Photograph (**a**) and line drawing (**b**) of the wing skeleton as well as enlarged images of the deltopectoral crest (**c**) and pneumatic foramen on the distal end of the wing metacarpal (**d**). Arrow points to the thin-section sample position on the first wing phalanx. *dc* deltopectoral crest, *dcs* distal carpals, *ep* unfused epiphyses, *hu* humerus, *ks* keratinous sheath of the second manual digit ungual, *mcI* metacarpal I, *mcI-III* metacarpals I–III, *md I* manual digit I, *md II* manual digit II, *mdIII* manual digit III, *pcs* proximal carpals, *pn* pneumatic foramen, *ra* radius, *se* carpal sesamoid, *ul* ulna, *wmc* wing metacarpal IV, *wp1* first wing phalanx, *wp2* second wing phalanx.

**Figure 2:**
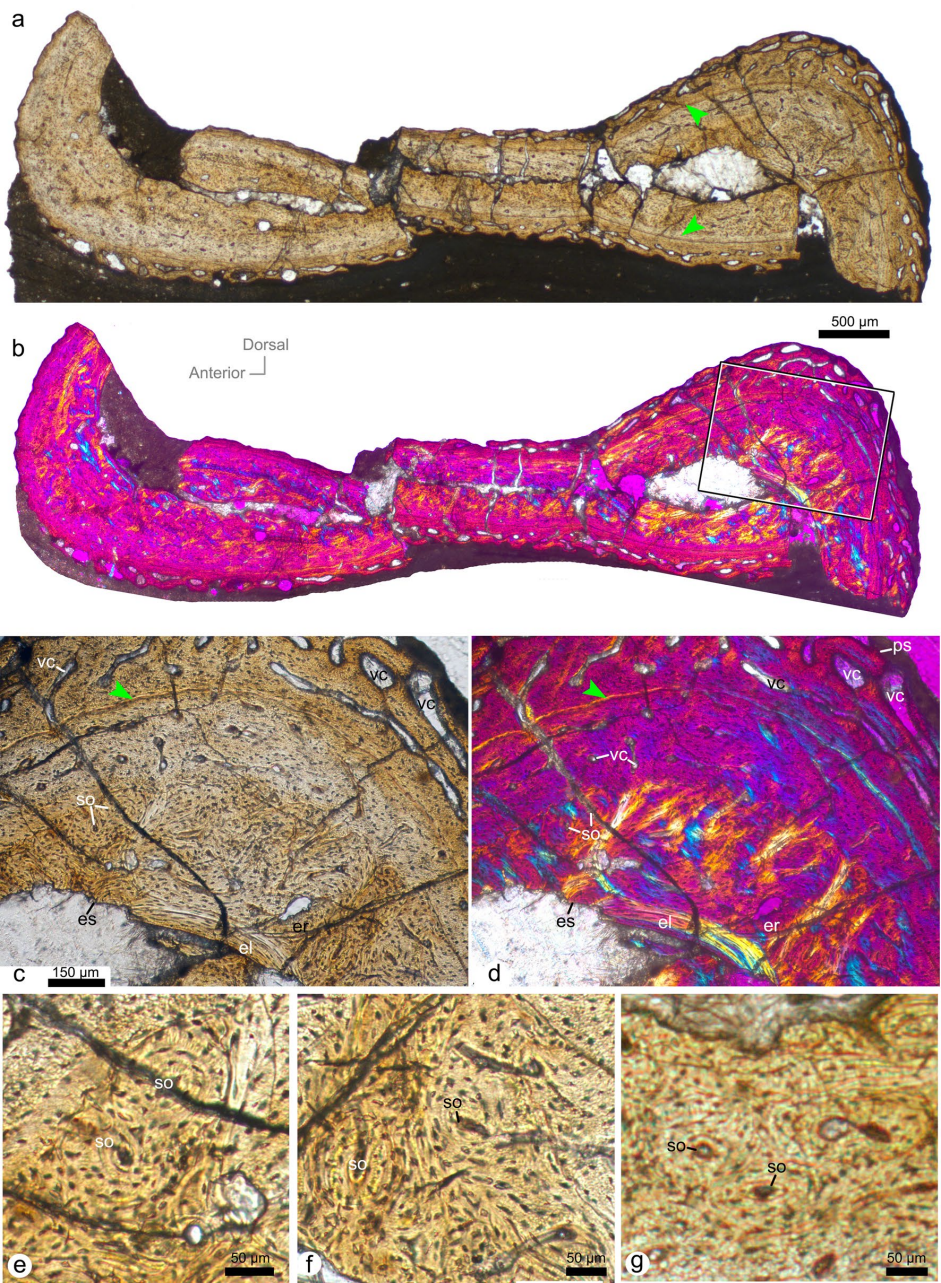
Histological thin-section of the first wing phalanx (SDUST-V1014; 71 µm in thickness). Whole cross-section (**a**, **b**) and enlarged image (**c**, **d**) under plain (**a**, **c**) and cross-polarized light (**b**, **d**), and higher enlarged images of secondary osteons under plain light (**e**–**g**). Arrow points to LAG. *el* endosteal lamella, *es* endosteal surface, *ps* periosteal surface, *so* secondary osteons, *vc* vascular canal.

**Figure 3:**
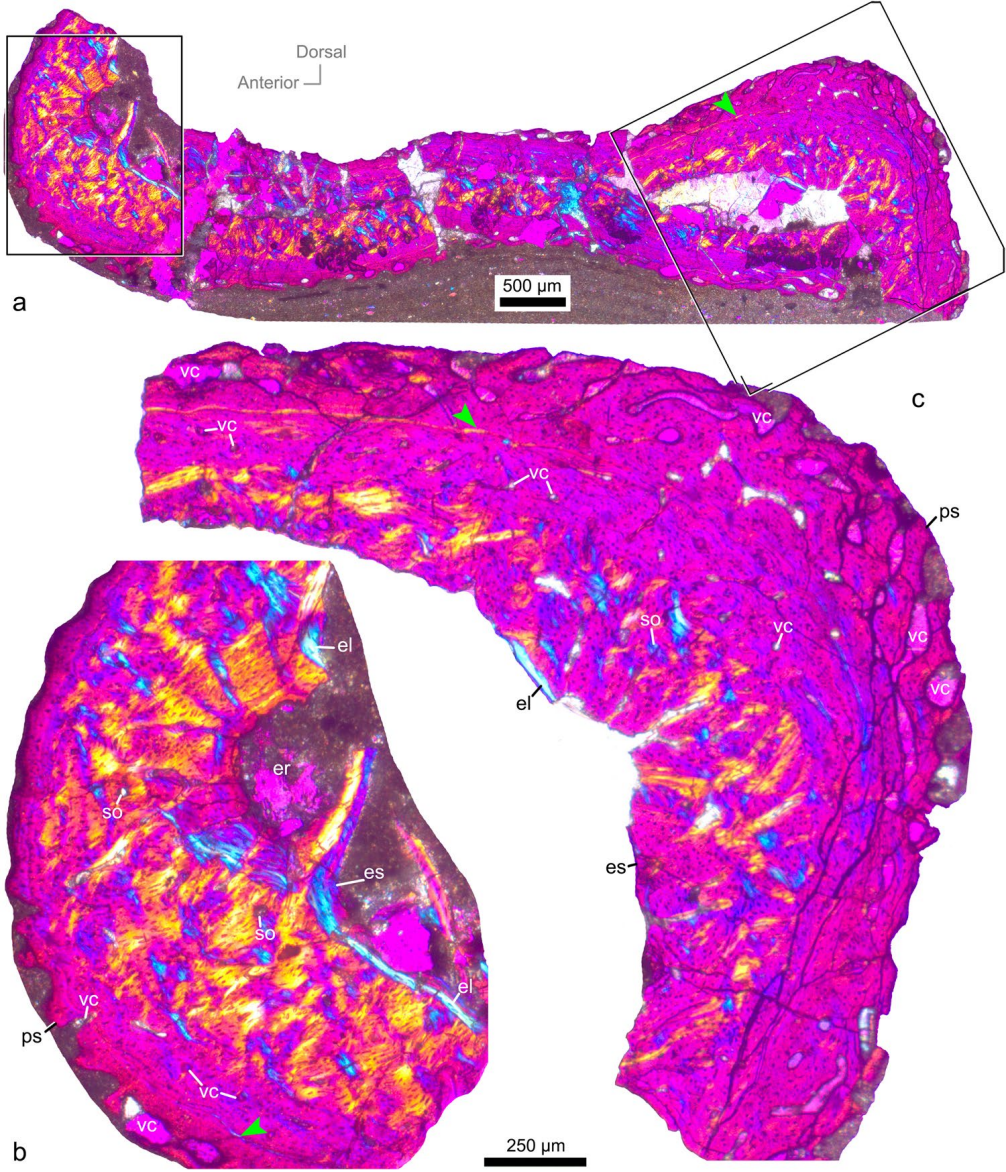
Histological thin-section of the first wing phalanx (SDUST-V1014; 30 µm in thickness). Whole cross section (**a**) and enlarged image (**b**, **c**) under cross-polarized light. Arrow points to LAG. *el* endosteal lamella, *er* erosion room, *es* endosteal surface, *ps* periosteal surface, *so* secondary osteon, *vc* vascular canal.

The new specimen (SDUST-V1014) is a left forelimb in articulation (Figs. 1, 2, 3) housed in the Vertebrate Palaeontological Collection, Shandong University of Science and Technology. It is a nearly complete wing skeleton, except for the missing two distal wing phalanges. The elements are strongly compressed in preservation and were exposed from one side by manual preparation under a Leica M165C microscope. Its measurements (See “Supplementary material”) was reported by Wu et al.^13^, but mislabeled as SDUST-V1004.

The fossil locality is at Liuligou Village, Sihedang Township, Lingyuan City, western Liaoning Province. Its lacustrine deposits are dominated by grey to grey-green shales and mudstones, belonging to the Early Cretaceous Jiufotang Formation^26^. The tapejarids “*Sinopterus lingyuanensis*” Lü et al.^27^ and “*Sinopterus atavismus*” Lü et al.^27^ have been reported from this area^6^.

### Comparative description

The left forelimb is about 511 mm in preserved length, lacking the length of the distal two wing phalanges. It is medium sized in relation to the other known Jehol tapejarids. Its bony elements are poorly ossified, such as the unfused epiphyses of humerus and ulna as well as the unfused bone complexes of the syncarpals and first wing phalanx-extensor tendon process. Based on Bennett’s^25,28^ size-independent criteria of pterosaurs ontogeny, these features indicate an immature stage for SDUST-V1014.

The humerus is exposed in ventral view and is about 73 mm in length. Proximally, the deltopectoral crest is well developed. It is plate-like, unwarped, and parallel-sided. Terminally, the crest is slightly expanded and forms a small distal prominence. This prominence is not known in other tapejarids^6,29–32^. The ulnar crest is damaged and lacks available information. Between these crests, the ventral surface of the humerus is concave and pneumatized. A pneumatic foramen is present at the level of the proximal margin of the deltopectoral crest, as in *Sinopterus dongi* (D3072)^7^, *Tapejara wellnhoferi* (SMNK PAL 1137)^29^ and *Tupandactylus navigans* (GP/2E 9266)^32^. The humeral shaft is nearly straight and slightly expanded distally; it is crushed and its surface is highly fractured. An unfused epiphysis is displaced slightly from the distal end of the humerus, positioned between the humerus distal end and the ulna proximal end.

The ulna and radius are elongate and straight, with the ulna much more robust and slightly longer than the radius. The ulna is 102 mm in length, whereas the radius is 100 mm in length. The ulna is expanded at the proximal and distal ends, and it partially overlaps the radius. An unfused epiphysis is articulated with the proximal end of the ulna by means of a distinct suture.

The carpus is partially disarticulated. Two proximal carpals, two distal carpals, a medial carpal, and a possible carpal sesamoid can be identified. The two proximal and distal carpals are not fused. This unfused condition is an immature feature found in pterosaurs^28,33^. Pneumatic foramina are visible on the distal carpals. A possible carpal sesamoid is articulated with the medial carpal.

The metacarpus is comprised of the slender metacarpals I–III and the robust wing metacarpal (IV). The metacarpals I–III are aligned parallel on the distal end of the metacarpus. The metacarpals II and III are much shorter than the metacarpal I. The metacarpal I is nearly complete and subequal to the metacarpal IV in length. The metacarpal I likely contacted the carpus, as in most Jehol tapejarids^3,6,7,34^. The metacarpal IV is about 103 mm in length and subequal to the ulnar length as in other tapejarids^13,32^ (See “Supplementary Material”). It is exposed in posterior view so that the pulley-like in shape of the two distal condyles is visible. These condyles are almost symmetric and separated by a broad intercondylar sulcus. The sulcus has a large pneumatic foramen at the proximal margin of the condyles (Fig. 1), as in *Sinopterus* (SDUST-V1012)^35^ and *Tapejara* (SMNK PAL 1137)^29^.

**Figure 4:**
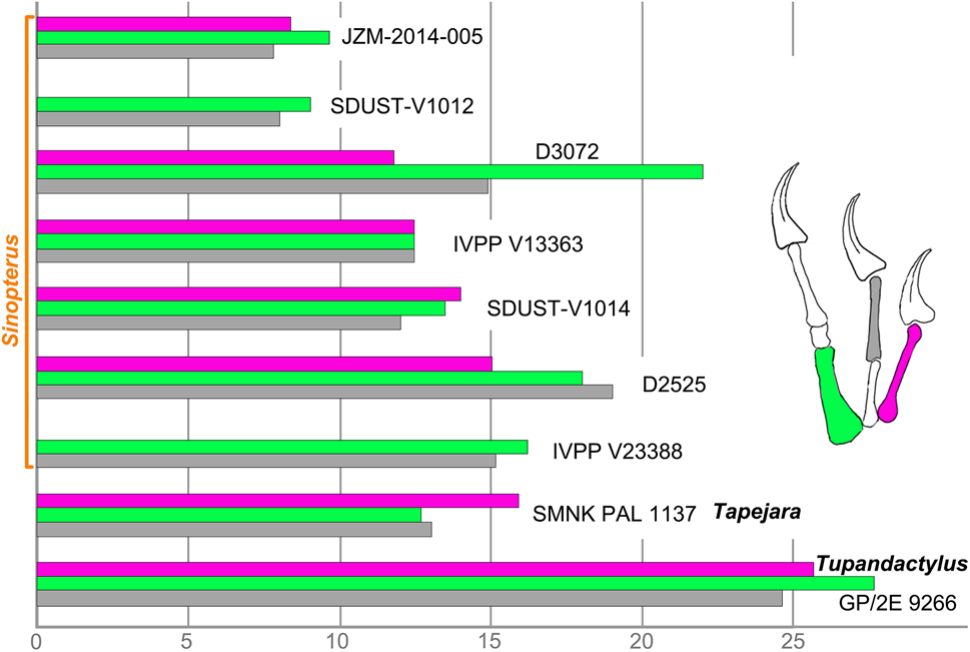
Comparisons of selected tapejarid manual phalanges. Phalangeal measurements are from this study and the literature^3,6,7,27,29,32,35,37^. Purple represents the manual phalanx I-1, green the manual phalanx III-1, and grey the manual phalanx II-2. Reconstruction of manual digits I–III is based on SDUST-V1014.

The manual digits I–III are completely preserved with the typical pterosaur phalangeal formula of 2–3–4 (Figs. 1 and 4). The phalanges are slender and rod-like, except for the squat phalanx III-2. The phalanx I-1 is the longest with a length of 14 mm. The phalanx III-1 has the second greatest length of 13.5 mm, but it is more robust than the other phalanges. The phalanges II-2 and III-3 have a subequal length of about 12 mm. The phalanx II-1 is relatively shorter with a length of 9 mm. The squat phalanx III-2 is the shortest with a length of 4 mm. These phalanges are expanded at the proximal and distal ends for their articular joints with adjacent elements. In digit II, the proximal phalanx II-1 is shorter than the penultimate phalanx II-2. The reverse condition is present in digit III, where the proximal phalanx III-1 is slightly longer than the penultimate phalanx III-3. These conditions are also known in some tapejarids (e.g., IVPP V13363^3^; JPM-2014-005^27^; and GP/2E 9266^32^). The first and second claws are well exposed in dorsal view, whereas the third claw is partially overlapped by the first wing phalanx. As in other tapejarids, the bony unguals are elongate and significantly curved. Proximally, the flexor tubercle extends anteriorly to about half the depth of the ungual proximal end. Distally, the ungual is curved to a sharp tip with a distinct midline groove. The first digit ungual appears to be more robust than the second, but its distal tip is broken off leaving a preserved length of about 11 mm. The second digit ungual is about 13 mm in length, measured directly from the proximoposterior end to the distal tip. Its curvature along the anterior margin is 114 degrees, similar to other tapejarids^13^. Distally, the keratinous sheath is preserved on the second digit claw, and it is about half the length of the bony ungual.

The proximal two of the four wing phalanges are well preserved in articulation, with the third and fourth phalanges entirely missing. The two preserved wing phalanges are exposed in dorsal view. Both are straight and highly elongated. The first wing phalanx is 134 mm in length, distinctly longer than the second phalanx’s 99.5 mm length. Proximally, the extensor tendon process is unfused to the first wing phalanx, an immature feature found in pterosaurs^25,28^, but together they form concavities to articulate with the wing metacarpal distal condyles. The proximal portion of the first wing phalanx is not pneumatized on the dorsal surface. The second wing phalanx is reduced in size relative to the first wing phalanx.

### Histological structure

**Figure 5:**
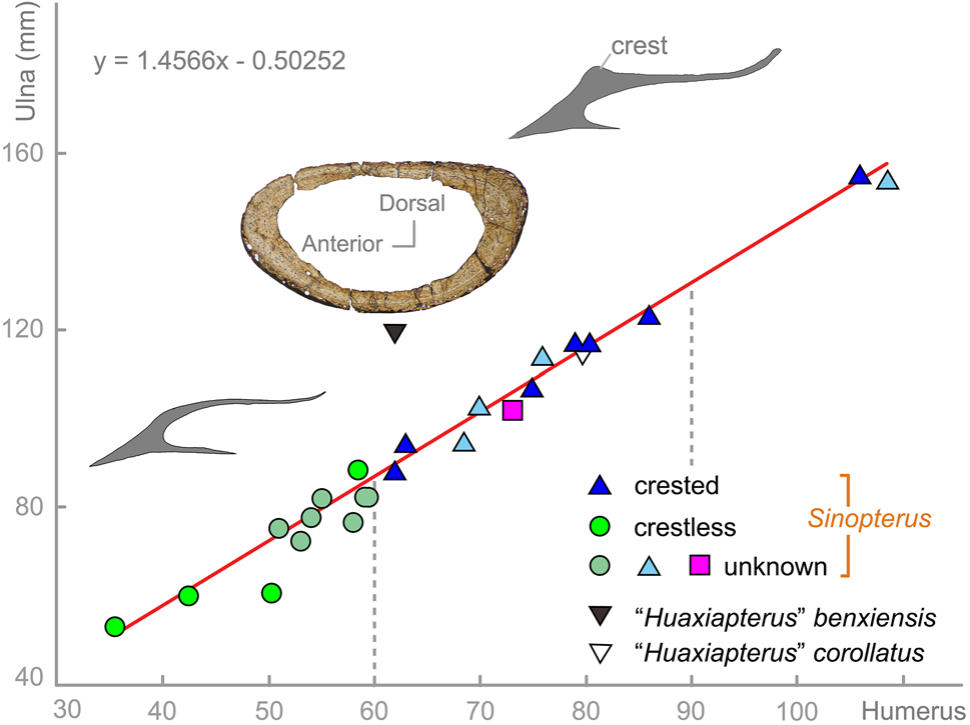
Plot of the ulna length relative to the humerus length in Jehol tapejarids with the presence of premaxillary crest indicated. Purple square represents SDUST-V1014. Histological cross-section reconstructed from the first wing phalanx of SDUST-V1014. Rostrum outlines are based on PMOL-AP00008 (crestless) and AP00025 (crested). Data provided in “Supplementary Material”.

Both thin-sections of SDUST-V1014 exhibit the transverse structure of the first wing phalanx well (Figs. 2 and 3). However, the bone wall is heavily crushed in the dorsoventral direction, with the dorsal and ventral walls contacting each other. Based on the broken portions, the transverse thin-section is reconstructed with an oval profile (Fig. 5). Its anteroposterior width is greater than its dorsoventral height, and its dorsal surface is flatter than the ventral surface. The bone wall varies from 0.4 to 1 mm in thickness, with the wall of the anterior and posterior ends distinctly thicker than that of the dorsal and ventral walls. The large internal cavity is hollow and lacks evidence of bony struts.

Histologically, the bone wall is highly-vascularized fibro-lamellar bone (Figs. 2 and 3). One line of arrested growth (LAG) is visible and indicates that the specimen was over a year in age when it died. The LAG is a thin brown line, more visible in thin-section with a thickness of 71 µm (Fig. 2). Around the cortex, it is close to the periosteal surface on the anterior end, but deeper on the posterior end. The cortex is subdivided by the LAG into inner and outer portions. The inner cortex is comprised of primary and secondary bones. The secondary bone is well developed around the marrow cavity. Its secondary osteons and parallel-fibered tissue are bright under cross-polarized light (Figs. 2 and 3). Most of the secondary osteons are arranged in oblique orientation. The osteocyte lacunae of the inner cortex are distinctly enlarged, in contrast to those in the outer cortex. Erosion rooms are identified in the secondary bone and vary in size. Of these, a large erosion room is present at the anterior corner of the thin-section (Fig. 3a,b). It has been opened onto the endosteal surface by damage to the endosteal lamella interpreted as a taphonomic artifact. The endosteal lamella (i.e., the parallel-fibered tissue) is locally developed at the anterior and posterior ends, where the maximum thickness of the cortex is reached. It is avascular as well as bright and band-like under cross-polarized light (Figs. 2b,c, and 3). Besides the lamella, the endosteal surface is coarse, representing the remodeling process. The primary bone is relatively thin and distributed along the LAG. Small vascular canals are mostly longitudinally directed. They are sparse in the dorsal and ventral walls but relatively dense in the anterior and posterior walls. The canals do not cross the LAG.

The outer cortex is distinctly thinner than the inner cortex. The vascular canals are enlarged and dominate the outer cortex. They are distinctly anastomosed and open at the periosteal surface, implying a high growth rate when it was alive. Whereas, the external fundamental system (EFS) is not developed in the outermost layer of the bony wall.

## Discussion

### Morphological comparisons of SDUST-V1014

As mentioned above, SDUST-V1014 shows an affinity with the Jehol azhdarchoids in having a plate-like and unwarped deltopectoral crest, reduced metacarpals II–III, and a second wing phalanx more reduced relative to the first wing phalanx. In the Jehol Biota, the azhdarchoids consists of two groups: chaoyangopterids and tapejarids. They are very different in cranial structure: a long and low rostrum in chaoyangopterids, or a short and tall rostrum in tapejarids. In addition, they are distinct from one another in proportions of the long bones^13^. The proportions of the forelimb elements in SDUST-V1014 match well with the tapejarids (Fig. 5)^13^.

In the Jehol Biota, the tapejarids are dominated by immature specimens. Their variation (e.g., cranial crest and limb proportions) has likely been over split and their diversity overestimated into nine nominal species^1,7,13,14^. In these varied features (e.g., cranial crest), ontogenetic variation of the cranial structures is well documented in some azhdarchoids (e.g., *Caiuajara* and *Tupuxuara*)^15,18^. Especially the premaxillary crest, which appears to vary with ontogeny and is associated with body size in Jehol tapejarids^6,7,12^. In addition, the Jehol tapejarids are all comparable in their postcranial skeleton and limb proportions, with the exception of “*Huaxiapterus*” *benxiensis* (Fig. 5)^1,13,14^. Therefore, a taxonomic revision has been proposed that most or even all the Jehol tapejarids should be synonymized as *Sinopterus*, but “*Huaxiapterus*” *benxiensis* needs to be further confirmed^1,7,8,12,14^. Based on the comparable limb proportions (wing metacarpal/humerus length ratio 1.41, wing metacarpal/ulna length ratio 1.01, wing metacarpal/first wing phalanx length ratio 0.77, and wing phalanges first/second 1.37)^7,13,14,35^, we refer SDUST-V1014 to *Sinopterus*.

In addition, the forelimb of SDUST-V1014 reveals more morphological information that adds to our knowledge of the Jehol tapejarids, such as the pneumatized humerus and wing metacarpal IV as well as the phalanges of manual digits I–III.

A pneumatized humerus is rarely found in the Jehol tapejarids^7^, but frequently found in Brazilian azhdarchoids such as *Caupedactylus*, *Keresdrakon*, *Tapejara*, *Tupandactylus*, and *Tupuxuara*^29,31,32,36^. Most of these bear a single pneumatic foramen on the ventral surface of the humeral head, whereas *Tapejara wellnhoferi* (SMNK PAL 1137)^29^ and *Tupandactylus navigans* (GP/2E 9266)^32^ are pneumatized on the dorsal and ventral surfaces of the humerus. In Jehol tapejarids, the dorsal surface of the humerus is more frequently exposed and lacks any evidence of pneumatic foramina^6,30,35,37^. By contrast, the ventral surface of the humerus is rarely exposed^7^. Therefore, it is uncertain how this ventral pneumatization of the humerus is distributed in the Jehol tapejarids.

The wing metacarpal is pneumatized posteriorly between the distal condyles in SDUST-V1014, as in *Sinopterus* (SDUST-V1012)^35^ and *Tapejara wellnhoferi* (SMNK PAL 1137)^29^. The opposite condition was reported in a Jehol tapejarid specimen (IVPP V 23388), in which the pneumatic foramen is certainly absent^6^. Due to crushed preservation, it is not known in the other Jehol tapejarids.

The manual digits I–III are well known in some Jehol tapejarids (e.g., D2525^37^; D3072^7^; IVPP V13363^3^; JPM-2014-005^27^). However, their phalangeal proportions appear to be variable (Fig. 4). For example, the phalanx III-1 is the longest in JPM-2014-005 and D3072; the phalanges I-1, II-2 and III-1 are subequal in IVPP V13363; and the phalanx II-2 is longer than I-1 and III-1 in D2525^37^. The new specimen SDUST-V1014 shows another distinct condition: the phalanx I-1 is the longest and the phalanx III-1 is second in length. The manual digits I–III are rarely found in other tapejarids, but two Brazilian tapejarids appear to have different phalangeal proportions as well: *Tapejara wellnhoferi* has a longer phalanx I-1 relative to III-1 (SMNK PAL 1137)^29^, and the opposite condition is present in the complete skeleton of *Tupandactylus navigans* (GP/2E 9266) in which the phalanx III-1 is longer than I-1^32^. However, this variation may be due to taxonomy or ontogeny and needs to be confirmed by additional material.

### Histological comparisons for ontogenetic stage

The histological structure of the first wing phalanx of SDUST-V1014 is characterized by high vascularity, an absence of the EFS, and vascular canals open on periosteal surface, which are well known in immature pterosaurs^24,25,28,38^. An immature stage for SDUST-V1014 is consistent with its skeletal fusion and histological structure. However, there are structures that are not typical for the juvenile stage of pterosaurs, such as secondary osteons well developed in the inner cortex, locally-distributed endosteal laminae, and the presence of a LAG. Especially, the presence of the fully-developed endosteal lamella indicates a termination of the marrow expansion, which is widely reported as the characteristic for the sexual maturity in pterosaurs^22–24,38,43^. In contrast, the endosteal lamella is developed locally at the anterior and posterior ends of the thin-sections of SDUST-V1014, possibly implying that the marrow cavity has begun to stop in expansion prior to the sexual maturity.

Furthermore, secondary osteons are poorly developed in pterosaurs^21,22,24,38–42^. However, dense secondary osteons have been observed in azhdarchids^38,43^. The presence of secondary osteons has also been reported in a wing metacarpal of the tapejarid *Caiuajara*, in which the specimen is mature as evidenced by fused scapulocoracoid and first wing phalanx-extensor tendon process^31,44^. In contrast, this condition is not present in the juveniles of the tapejarid *Keresdrakon*^31,45^.

The presence of one LAG indicates the individual was over a year in age when it died. In general, pterosaurs have a fast growth rate early in ontogeny, lasting for the first 2–3 years^21–23,25,42^. During this stage, the histological structure is characterized by well-vascularized fibrolamellar bone. Afterwards, it turns into poorly-vascularized lamellar bone with a slower growth rate at the onset of sexual maturity^21,22,25,42^. As described above, the thin-sections of SDUST-V1014 match well with an early ontogenetic stage. The one visible LAG suggests that the specimen had reached the last year of the early ontogenetic stage and was closer to the sexual maturity.

Furthermore, vascular canals open on the periosteal surface of the bone is typical for pterosaur individuals from juvenile to subadult stage^23,24,28,31^. Based on these features, SDUST-V1014 is likely a later juvenile prior to the sexual maturity.

### Development of the premaxillary crest

The Jehol tapejarids plot as a distinct ontogenetic series in size, except for “*Huaxiapterus*” *benxiensis* (Fig. 5)^13,14^. Their cranial crests, especially the presence of a premaxillary crest, is correlated with size as exemplified by the plot of the humerus and ulna in length (Fig. 5). The crestless condition is only present in the small-sized individuals in which the humeral length is less than 60 mm. The crested condition occurs in the larger individuals. Furthermore, most of the crested individuals are medium in size, forming a range of humeral length from 62 to 86 mm. These medium-sized individuals are immature in skeletal development^7,12,14^. In contrast, the two rare adults at skeletal maturity (106.0 and 108.5 mm in humeral length) are separated from the medium-sized individuals by a size gap. One of the two adults (PMOL-AP00011-1) is well preserved with a crested skull, but it remains undescribed.

The new specimen (SDUST-V1014) falls well into the medium-sized range of the crested individuals. Its histological information provides a good reference for this medium-sized group. The group is also at the high-speed growth prior to sexual maturity, although its upper end might reach the sexual maturity. Therefore, the premaxillary crest occurs in this group perhaps as an ontogenetic feature, rather than just a sexually dimorphic and/or taxonomic feature. Furthermore, the premaxillary crest appears to be variable in size and shape among the medium-sized individuals, possibly representing individual and/or ontogenetic variation as in the Brazilian tapejarid *Caiuajara*^15^. Therefore, any further study should be cautious about the variation of the premaxillary crest with respect taxonomy and diversity at this stage.

### Ecological implications for the Jehol tapejarids

As discussed above, immature Jehol tapejarids are at the high-speed growth stage, and the largest immature individuals might have reached sexual maturity. In contrast, only two adults at skeletal maturity have been found (See “Supplementary Material”)^13,37^. These are comparable in size, possibly representing the largest size at skeletal maturation. Generally, sexual maturity occurs earlier than skeletal maturity in pterosaurs^21–23,25,42^. However, this ontogenetic strategy appears to vary in different taxa. For instance, sexual maturity occurs at about half of the largest adult size of the skeletal maturity in *Pterodaustro*^21,22^, whereas sexual maturity occurs at about 80% of the largest adult size of the skeletal maturity in *Pteranodon*^25^. The largest immature specimen of *Sinopterus* (PMOL-AP00013, “Supplementary Material”) is about 79% of the largest adult wing-span (D2525; calculated from the data in Wu et al.^13^). Therefore, the ontogeny of Jehol tapejarids is possibly comparable with that in *Pteranodon* and different from *Pterodaustro*. Furthermore, similar ontogeny seems to be present in the Brazilian tapejarid *Caiuajara* in which a humerus of juveniles is 79% the length of the adult humerus at the skeletal maturity (calculated from the data in Manzig et al.^15^), although this proposal currently lacks histological evidence.

As reviewed by Bennett^25^ and Naish et al.^14^, the ontogeny of pterosaurs is variable. For instance, pterosaurs such as *Rhamphorhynchus* and *Pterodactylus* are missing sexually mature individuals in their fossil assemblages. Abundant immature individuals and rare adults at skeletal maturity imply that the latter has a different habitat elsewhere. The pteranodontians (e.g., *Pteranodon*, *Nyctosaurus*, and *Anhanguera*) are dominated by mature individuals and are missing juveniles. Whereas the ontogenetic series is nearly complete in filter-feeding ctenochasmatids (e.g., *Ctenochasma* and *Pterodaustro*), implying they have a similar habitat^21,25^. In the Jehol tapejarids, sexually mature individuals appear to be almost absent in the ontogenetic series (Fig. 5). This occurrence is likely not a taphonomic or collection bias because the adults at skeletal maturity are still rarely recorded and larger specimens tend to be preferred for collections. Instead, the lack of the sexually mature individuals likely indicates a habitat difference between the sexually mature and immature individuals, as in *Pterodactylus*^25^ and *Rhamphorhynchus*^46^. A similar condition is possibly present in *Caiuajara*, in which juveniles or very young are predominant, but the adults at skeletal maturity are quite rare^15^. However, *Caiuajara* was considered to be a resident pterosaur, although the possibility of a migratory habit was also hypothesized^15^. A migratory habit seems to be more suitable to explain the lack of subadult individuals at sexual maturity in *Caiuajara* and the Jehol tapejarids, in combination with their herbivorous habits^11,13^. Therefore, the Jehol Biota is hypothesized to be a breeding area, at least for the tapejarid pterosaurs. Upon reaching sexual maturity, the tapejarids would disperse and return to reproduce when they had reached full skeletal maturation, similar like extant migratory birds.

## Methods

### Histological thin-sections

Histological thin-sections of SDUST-V1014 were performed at the histological lab of the Key Laboratory of Vertebrate Evolution and Human Origins, IVPP. Two transverse sections of 71 µm (Fig. 2) and 30 µm (Fig. 3) in thickness were taken from the first wing phalanx using standard paleohistological techniques of Padian and Lamm^47^. They were photographed under normal and cross-polarized light with a Leica DM4 P microscope.

### Institution abbreviations

D, Dalian Natural History Museum (Dalian, China); GP/2E, Laboratório de Paleontologia Sistemática do Instituto de Geociências da Universidade de São Paulo (São Paulo, Brazil); IVPP, Institute of Vertebrate Paleontology and Paleoanthropology, Chinese Academy of Sciences (Beijing, China); JPM, Jinzhou Paleontological Museum (Jinzhou, China); PMOL, Paleontological Museum of Liaoning, Shenyang Normal University (Shenyang, China); SDUST, Shandong University of Science and Technology (Qingdao, China); SMNK, Staatliches Museum für Naturkunde (Karlsruhe, Germany).

## Supplementary Information

Supplementary Information.

## Data Availability

The published article includes all the data generated in the text. The measurements of the Jehol tapejarids can be found in the supplementary information.
