# Supplementary material for: A new wing skeleton of the Jehol tapejarid Sinopterus and its implications for ontogeny and paleoecology of the Tapejaridae
Source: Sci Rep. 2022 Jun 17;12:10159. doi: 10.1038/s41598-022-14111-2 (PMC9205892; doi:10.1038/s41598-022-14111-2)
Supplement: Supplementary file 1 — Supplementary Information. [file 41598_2022_14111_MOESM1_ESM.pdf]

A new wing skeleton of the Jehol tapejarid *Sinopterus* and its implications for ontogeny and paleoecology of the Tapejaridae

Chang-Fu Zhou[1*], Dongxiang Yu[1], Ziheng Zhu[1], Brian Andres[2]

[1]College of Earth Science and Engineering, Shandong University of Science and Technology, Qingdao, Shandong Province, China.

2 Department of Health, University College Birmingham, Birmingham, U.K.

Correspondence and requests for materials should be addressed to C.-F.Z. (zhoucf528@163.com).

Table 1. Measurements of the Jehol tapejarids.

	Hu	Ul/Ra	Wmc	Wp1	Wp2	Premaxillary crest	Citation
***Sinopterus dongi* = *S. “gui”* (BMNHC-PH077 = BPV-077)**	35.5	52.9	>50	56	?	no	Li et al. 2003
***Sinopterus atavismus* (XHPM 1009)**	42.4	59.9	63	80.7	63.6	no	Lu et al. 2016
***Sinopterus lingyuanensis* (JPM-2014-005)**	50.3	60.1	60.2	83.6	66.9	no	Lu et al. 2016
**Jehol tapejarid (PMOL-AP00025)**	51	76	87	114	81	?	Wu et al. 2017
**Jehol tapejarid (PMOL-AP00021)**	53	73	?	?	?	?	Wu et al. 2017
**Jehol tapejarid (SDUST-V1013)**	54	78	90	107	80	?	Wu et al. 2017
***Sinopterus dongi* (DLNHM D3072)**	55.1	82.6	90.8	108.2	85.6	?	Shen et al 2021
**Jehol tapejarid (PMOL-AP00016)**	58	77	85	104	83.5	?	Wu et al. 2017

***Sinopterus dongi* (IVPP V13363)**	58.5	88	95	121	89.5	no	Wang and Zhou 2003
**Jehol tapejarid (PMOL-AP00022)**	59	82	88	122	82	?	Wu et al. 2017
**Jehol tapejarid (PMOL-AP00007)**	59.5	82	96	120	94	?	Wu et al. 2017
**Jehol tapejarid (PMOL-AP00017)**	62	88	100	125	?	yes	Wu et al. 2017
***"H." benxiensis* (BXGM V0011)**	62	119	133	176	130	yes	Lü et al. 2007
***Sinopterus dongi* = *"Eopteranodon lii"* ((BMNHC-PH078 = BPV 078)**	63	94	99.5	131	99	yes	Lü & Zhang, 2005
***Sinopterus dongi* = *"Eopteranodon lii"* (DLNHM D2526)**	68.5	94.5	105.5	135.5	102.5	?	Lü, Gao et al. 2006
**Jehol tapejarid (LPM-L111609)**	70	103	105	137	?	?	Wu et al. 2017
**Jehol tapejarid (SDUST-V1014)**	73	102	103	134	99.5	?	Wu et al. 2017
**Jehol tapejarid (PMOL-AP00009)**	75	107	108	145	106	yes	Wu et al. 2017
**Jehol tapejarid (PMOL-AP00030)**	76	114				?	Liu et al. 2015
***Sinopterus dongi* = *"Huaxiapterus jii"* ((CDL)KLY-HXYL = "GMN"-03-11-001)**	79	117	132	161.5	127	yes	Lü & Yuan, 2005
***"H." corollatus* (ZMNH M8131)**	79.7	114	152	176	108.5	yes	Lü, Jin et al. 2006
***Sinopterus atavismus* (IVPP V23388)**	80.4	116.8	126.4	159.4	123.2	yes	Zhang et al. 2019
**Jehol tapejarid (PMOL-AP00013)**	86	123.5	117	175	127	yes	Wu et al. 2017
**Jehol tapejarid (PMOL-AP00011-1)**	106	155	160	?	150	yes	Wu et al. 2017
***Sinopterus dongi* (DLNHM D2525)**	108.5	154	169.5	215	156	?	Lü, Liu et al. 2006

Abbreviations: Hu, humerus; Ra, radius; Ul, ulna; Wmc, wing metacarpal; Wp1, first wing phalanx; Wp2, second wing phalanx. Measurements (mm) from the literature.
